# ‘They Were Talking to Each Other but Not to Me’: Examining the Drivers of Patients' Poor Experiences During the Transition From the Hospital to Skilled Nursing Facility

**DOI:** 10.1111/hex.70248

**Published:** 2025-04-28

**Authors:** James D. Harrison, Margaret C. Fang, Rebecca L. Sudore, Andrew D. Auerbach, Tasce Bongiovanni, Audrey Lyndon

**Affiliations:** ^1^ Division of Hospital Medicine University of California San Francisco San Francisco California USA; ^2^ Division of Geriatrics University of California San Francisco San Francisco California USA; ^3^ Department of Surgery University of California San Francisco San Francisco California USA; ^4^ New York University Rory Meyers College of Nursing New York New York USA

**Keywords:** care transitions, grounded theory, hospitalisation, skilled nursing facilities

## Abstract

**Introduction:**

Hospital‐to‐skilled nursing facility (SNF) transitions have been characterised as fragmented and having poor quality. The drivers, or the factors and actions, that directly lead to these poor experiences are not well described. It is essential to understand the drivers of these experiences so that specific improvement targets can be identified. This study aimed to generate a theory of contributing factors that determine patient and caregiver experiences during the transition from the hospital to SNF.

**Methods:**

We conducted a grounded theory study on the Medicine Service at an academic medical centre (AMC) and a short‐term rehabilitation SNF. We conducted individual in‐depth interviews with patients, caregivers and clinicians, as well as ethnographic observations of hospital and SNF care activities. We analysed data using dimensional analysis to create an explanatory matrix that identified prominent dimensions and considered the context, conditions and processes that result in patient and caregiver consequences and experiences.

**Results:**

We completed 41 interviews (15 patients, 5 caregivers and 15 AMC and 6 SNF clinicians) and 40 h of ethnographic observations. ‘They were talking to each other, but not to me’ was the dimension with the greatest explanatory power regarding patient and caregiver experience. Patients and caregivers consistently felt disconnected from their care teams and lacked sufficient information leading to uncertainty about their SNF admission and plans for recovery. Key conditions driving these outcomes were patient and care team processes, including interdisciplinary team‐based care, clinical training and practice norms, pressure to maintain hospital throughput, patient behaviours, the availability and provision of information, and patient's physical and emotional vulnerability. The relationships between conditions and processes were complex, dynamic and, at times, interrelated.

**Conclusion:**

This study has conceptualised the root causes of poor‐quality experiences within the hospital‐to‐SNF care transition. Our theory generation identifies targets for clinical practice improvement, tailored intervention development and medical education innovations.

**Patient or Public Contribution:**

We partnered with the Hospital Medicine Reengineering Network (HOMERuN) Patient and Family Advisory Council during all stages of this study.

## 1 Introduction

Millions of Americans are discharged annually from a hospital to one of almost 15,000 skilled nursing facilities (SNFs) [1]. In the United States, SNFs provide temporary transitional care for people who require rehabilitation and medical treatments after hospitalisation for an illness, injury or chronic condition [2]. The primary goal of short‐term rehabilitation at SNFs is for an individual to regain function and independence so that they can safely return home [3]. The prevalence and costs associated with this care transition will rapidly increase as the United States population ages [4].

Hospital‐to‐SNF transitions have previously been characterised as fragmented and ones that are marked by poor communication between patients, caregivers, hospital and SNF clinicians [5, 6, 7]. This results in gaps in hospital discharge planning, SNF admission processes and an overall poor‐quality transition experience [8, 9, 10, 11, 12]. This care transition can be a fraught time; studies have documented that patients are often inadequately prepared for discharge and unprepared for SNF care and their post‐acute care recovery [13, 14]. This care transition causes considerable distress for patients and their caregivers [6, 15, 16].

Despite how common this care transition is and the consistent poor patient experiences reported, most research seeking to improve care transitions has focused on hospital discharges to home rather than to SNFs. Research that does exist focuses primarily on describing the experiences of either patients, caregivers or clinicians from their individual perspectives [5, 7, 9, 10, 15]. What has not been as well examined are the *drivers* of these experiences and how these *drivers* of experiences are influenced by patients, caregivers and clinicians concurrently within the hospital and SNF setting. Drivers are defined as the factors and actions that directly lead to the experiences and outcomes [17] of patients and caregivers during this care transition. It is essential to understand the drivers of patients' and caregivers' hospital‐to‐SNF transition experiences so that specific improvement targets can be identified and gaps addressed. Only then can the provision of high‐quality, patient‐centred care—which is a national and patient priority—be realised [18, 19, 20, 21]. Therefore, the aim of this study was to generate a theory of contributing factors that determine patient and caregiver experiences during the transition from the hospital to SNF.

## 2 Materials and Methods

### 2.1 Approach

We used the theory‐methodology package of symbolic interactionalism and constructivist grounded theory to guide our study [22, 23]. This study was approved by the University of California San Francisco Institutional Review Board (IRB). We partnered with a Patient and Family Advisory Council (PFAC) [24] during all stages of this study.

### 2.2 Setting

Our study took place in the Medicine Service at a large quaternary academic medical centre (AMC) and an SNF both located in San Francisco, the United States. The Medicine Service includes 8 care teams (comprising hospitalist physicians, resident physicians and students) and 10 direct care hospitalist‐only services. The SNF is a post‐acute rehabilitation facility with approximately 90 beds for temporary short‐term stays. Other care team members at both sites are nurses, case managers and physical and occupational therapists. See Appendix 1 for more details about study sites and the care delivered.

### 2.3 Participants

Patient participants were eligible for interview if they were ≥ 50 years old, English‐speaking without dementia and admitted to the SNF within 7 days from the AMC. We excluded patients ≤ 49 years as they are less likely to be admitted to SNFs [1]. People with Alzheimer's and related dementias (ADRD) were also excluded to maintain a more homogenous study sample and given the unique needs of these specific patient groups. Caregivers of eligible patients were also eligible. Patient and caregiver participants were eligible for ethnographic observations if they were ≥ 18 years old and admitted to the Medicine Service or SNF. All adults were included in observations to ensure the feasibility of conducting observations in a busy hospital and SNF setting. Clinicians included hospital and SNF physicians, nurses, case managers and physical and occupational therapists.

### 2.4 Recruitment

We utilised two sampling methods consistent with grounded theory, purposive and theoretical [22]. Firstly, we screened the SNFs' Electronic Health Record (EHR) to purposefully identify eligible patients and their caregivers who were then invited to participate in an interview during their SNF admission. A SNF staff member—not involved in the patient's care—approached patients and asked if they were interested in learning more about the study. If they were, a member of the research team would provide study information. Similarly, patients and caregivers were asked by a staff member not involved in their care before inviting them to be observed during care interactions. Purposive sampling was also used to invite clinicians by email and staff meetings to participate in interviews and observations. Once data collection had commenced, we used theoretical sampling to make additional recruitment decisions to further explore the range, variation and inter‐relationships of concepts from developing data analysis [22]. As approved by our IRB, all participants provided written informed consent for interviews and verbal informed consent for observations.

### 2.5 Data Collection

The lead author conducted in‐depth face‐to‐face interviews at the SNF and ethnographic participant observations at the hospital and SNF. An interview guide was developed to explore participants' experiences and reflections on the hospital‐to‐SNF transition, focusing on discharge planning, expectation setting, the transition process, SNF admission and post‐hospital recovery (Appendix 2). Interviews were 45–60 min long and were digitally recorded and professionally transcribed. Ethnographic observations were embedded into day‐to‐day care activities, unobtrusively observing and taking notes of hospital and SNF care team processes and interactions between clinicians, patients and caregivers (e.g., communication, teamwork and non‐verbal cues) [25, 26, 27]. Observations occurred during the daytime (morning ward rounds, family meetings, interprofessional discharge meetings, SNF admissions and routine clinical care delivery). We did not observe at night, given transition planning and admission to SNF mainly occur during the day. To facilitate accurate recollection, following interviews and observations, we used guidelines for structuring field notes and memos [28, 29]. Data collection and analysis continued until theoretical saturation—the point at which additional data collection yielded no new properties or theoretical insights—was achieved [22].

### 2.6 Analysis

Data were collected and analysed simultaneously using constant comparison and dimensional analysis [22, 30, 31]. A defining feature of dimensional analysis is the creation of an explanatory matrix, which explores the prominent dimensions in the data and determines a central perspective from which to organise other key dimensions within the matrix's categories (i.e., context, conditions, processes and consequences) [32, 33]. Context represents the setting or environment in which the phenomenon is embedded. Conditions facilitate, impede or influence the participants' central actions. Processes are actions shaped by the conditions, and consequences are the outcomes resulting from these processes.

The first step of analysis involved dimensionalizing, a type of open coding that summarises meanings or actions [30]. Codes were used to provisionally label dimensions and aspects of their properties. Initial codes included sensitising concepts [34], which were existing reported problems related to SNF transitions (e.g., feeling unprepared [16]), as well as codes that were unique to the data. This was then followed by designation which expanded the analysis by exploring the breadth of dimensions of experience without consideration of their importance until sufficient dimensions were identified to represent the emerging concepts [30, 31]. Sensitising concepts only remained if they were grounded in data [34]. Using analytic memos and team analysis meetings, dimensions were then differentiated by considering their relative significance until we distinguished a critical mass of codes that best described the salient dimensions of experience. Relationships between dimensions were then explored by creating an explanatory matrix, where several plausible dimensions were ‘auditioned’ for their fit as key dimensions that explained the central perspective [30, 31]. The initial goal when using the matrix was to hold possibilities open by giving several key dimensions an opportunity to serve as the central perspective. This central perspective enabled the integration of other key dimensions within the matrix according to their fit, such as context, conditions, processes and consequences. Data were managed using ATLAS.ti 9.0, and analysis was conducted by authors J.H., T.B. and A.L. We took several steps to ensure theoretical and methodological rigour and credibility throughout the analysis, including sustained immersion in the field, ensuring multivocality, writing memos on individual interviews and dimensions, triangulation of data sources, and frequent meetings with team members for analysis development and direct exploration of reflexivity and positionality [22, 35, 36].

## 3 Results

### 3.1 Participants and Explanatory Matrix

**Figure 1:**
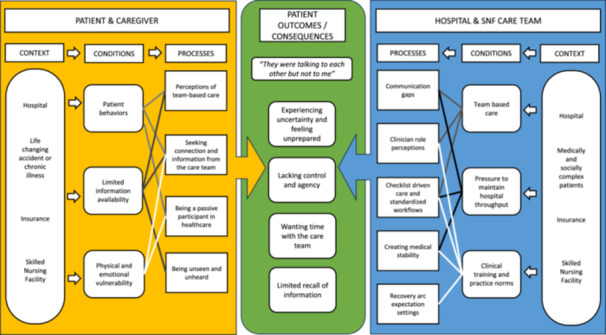
Theoretical framework and explanatory matrix describing the drivers (context, conditions and processes) of patient experiences (outcomes/consequences) during the hospital‐to‐SNF care transition.

We completed 41 interviews (15 patients, 5 caregivers and 15 hospital and 6 SNF clinicians) and 40 h of ethnographic observations (30 in the hospital and 10 in the SNF). Study participants' characteristics are shown in Table 1. We configured the explanatory matrix around the central perspective: ‘They were talking to each other, but not to me’ given patients and caregivers consistently noted they felt disconnected from their care team, lacked sufficient information, and experienced uncertainty about their admission to an SNF and long‐term recovery. In Figure 1, we visualise this matrix and generate a theory detailing the context and conditions that drive key processes and actions of patients, caregivers and clinicians that cause the challenges experienced by patients and caregivers during this care transition. The inclusion of patients, caregivers and clinicians in our matrix acknowledges their combined influences and interactions, which create the conditions and processes that result in the consequences experienced by patients. While we have delineated conditions and processes for reporting purposes, the relationships between some conditions overlap and impact several processes (Figure 1). Additional representative quotes and observation field notes related to the conditions, processes and consequences identified can be found in Appendix 3.

**Table 1:** Study participant demographic, clinical and practice characteristics.

	Patients (*n* = 15)	Caregivers (*n* = 5)
Mean age (SD, range)	67.3 (8.0, 54–78)	—
Gender		
Female	10 (67)	3 (60)
Male	5 (33)	2 (40)
Race		
African American/Black	1 (7)	—
Other	3 (20)	—
White	11 (73)	5 (100)
Ethnicity		
Hispanic or Latino	2 (13)	
Not Hispanic or Latino	13 (87)	5 (100)
Insurance		
Medicare	12 (80)	—
Private	3 (20)	—
Mean length of hospital stay in days (SD, range)	11.8 (10.3, 3−35)	—

	**Clinicians**	
	**Hospital (*n*** = **15)**	**SNF (*n*** = **6)**
Clinician type		
Hospitalist	6 (40)	1 (17)
Geriatrician	1 (7)	3 (50)
Registered nurse	2 (13)	—
Nurse practitioner	1 (7)	1 (17)
Case manager	3 (20)	1 (17)
Physical therapist	1 (7)	—
Occupational therapist	1 (7)	—

### 3.2 Context: Life Changing Accidents and/or Chronic Illness; Healthcare Settings; Medically and Socially Complex Patients and Health Insurance

An accident and/or chronic illness, two *healthcare settings* (a hospital and a SNF), medically and socially complex patients, and health insurance formed the boundaries of our enquiry and the environment in which the dimensions in our explanatory matrix occurred. Patients' *hospitalisation and SNF* admission were caused by a *life‐changing accident or a chronic illness*. Hospitalisation then created or accelerated pre‐existing functional limitations that led to physical deconditioning that required post‐acute care rehabilitation at SNF. Each hospital and SNF clinician described patients with high acuity, multiple comorbidities and who were *medically and socially complex*. *Healthcare insurance* was an omnipresent factor that shaped this care transition. Insurance coverage to pay for SNF care depended on the type of policy and the nature of the care required. Insurance had complex eligibility requirements that determined the eventual patient's SNF location. Patients and caregivers were given a list of available SNFs based on their insurance status—better insurance resulted in more available options.

### 3.3 Patient and Caregiver Conditions: Patient Behaviours; Limited Information Availability and Physical and Emotional Vulnerability

*Patient and caregiver behaviours* and *limited information availability* were distinct but related conditions impacting key transition processes and drove how and why patients and caregivers responded to illness and engaged in care. These behaviours were often a result of whether information was made available or not to them. The delivery of verbal information regarding hospital discharge planning and SNF admission typically occurred during in‐person interactions with care team members. Written information was provided in a hospital discharge summary or within an admissions folder located in each patient's room at the SNF. Information, though, was delivered inconsistently in each care setting. The final conditions were *physical and emotional vulnerability*. Hospitalisation and post‐acute care exposed the vulnerability of physical health, while the emotional vulnerability was a result of the unfamiliar clinical environments that patients had entered to receive care and rehabilitation.

### 3.4 Patient and Caregiver Processes and Actions

There were four key processes and actions of patients and caregivers. The conditions previously described either independently or, in some instances, in combination, shaped these processes and actions.

#### 3.4.1 Perception of Team‐Based Care

Patients and caregivers lacked understanding and were confused by team‐based care leading to behaviours where they would exclusively focus on the information provided by physicians. They could not recall discussions with other clinicians. Patients' and caregivers' view of clinical care reflected physician‐led care and not team‐based care. Despite patients and caregivers not recalling information given to them by other care team members, case managers were observed to have a clear process for partnering with patients during discharge planning.

‘From the start to finish we gather information about the patient's clinical care and readiness for discharge. Then we do a Case Management Assessment…their demographics, where they live, who their support system is, what services may have already been set up for them, as well as reviewing insurance information. We then ask them what they foresee as their goal for discharge and what they want to do based on the recommendations that we are seeing in the hospital. Then we work with the patient to achieve this’.

Hospital case manager

Confusion with team‐based care impacted patients' and caregivers' ability to retain information. In many instances they were unable to distinguish the large number of clinicians entering their room. This was further hindered because everyone wore similar coloured scrubs or masks. While care team members would introduce themselves by name and role, this often did not improve patients' ability to understand.

‘It's very confusing to be in the hospital. There are three teams that were dealing with me – the medical team, the surgical team, and I guess the other people who take care of me, whatever that's called. I had people coming in and out. I didn't know who was who, or whom I was supposed to talk to. I didn't know who was in charge.’

Patient

#### 3.4.2 Being a Passive Participant in Healthcare

The most common patient behaviour observed was being a passive participant in their healthcare. This was not a choice but a behaviour rooted in the belief of how the patient–clinician relationship should be. It was also a result of the fact that patients and caregivers were often not invited to share their priorities. Many patients described how they did their best to be ‘a very good patient’ or explained that ‘I'm an easy patient to be around’, so they were surprised that information about the SNF and their recovery trajectory was not forthcoming. Others noted that they ‘kept quiet’ or did not want to ask questions as they ‘did not want to be burden on their care team, or slow down them down as they were already busy enough and overextended’.

#### 3.4.3 Being Unseen and Not Heard by the Healthcare Team

A combination of patient behaviours (e.g., being a passive participant) and limited information provision led to patients' feeling unseen and not heard. Bedside rounds were a specific time during hospitalisation when patients hoped they would be able to connect with physicians. However, bedside rounds were time‐limited and left patients feeling unseen, unheard and without the information they wanted. For example, one patient did not want to be in the hospital for her birthday and had questions about discharge options. However, the team was unable to answer all her questions as they needed to keep moving and see other patients. The team began to edge out of the room, taking steps backwards to slowly move away from the patient as she continued her conversation, peppering them with questions. This process was repeated throughout rounds and reflected the lack of time available to develop connections in a way patients desired. Many patients and caregivers felt part of a clinical process that did not consider their individuality outside of their medical or rehabilitation needs.

‘I think sometimes we focus on we're going to transfer you in a wheelchair van, and we're going to get you there. When you get there, they're going to do an assessment, you'll meet with their physical therapist and occupational therapist. I feel like that kind of stuff is very boilerplate because those are requirements that they're legally bound to be covered by insurance. But the stuff that makes the real difference in the quality of the patient's overall well‐being is holistically looking at the patient. Do we look at the holistic, the non‐regulated pieces and look at the overall quality and standard of care being given?’

Caregiver

#### 3.4.4 Seeking Connections and Information From the Healthcare Team

The lack of information provision, patients' vulnerability and feelings of not being seen and heard forced some to seek connections with the healthcare team. Sometimes, this was to have a clinical need addressed or for specific information, but mainly they wanted a personal connection so that the team could understand their perspective and needs.

‘I wish I had more communication with them in the hospital because they weren't understanding all the pain I was having. I understand that they have more than one patient, so I was putting that in my mind that there's a lot of patients there. I wish I could have had more time with a doctor or nurse and tell them what's going through my mind, but I didn't have that much time with them.’

Patient

### 3.5 Clinician Conditions: Team‐Based Care; Pressure to Maintain Hospital Throughput; Clinical Training and Practice Norms

Team‐based care influenced clinician behaviour and practice. As noted, team‐based care also had direct implications for patients, impacting their ability to retain information due to confusion and misunderstanding of this structure of care. Hospital teams noted the *pressure to maintain hospital throughput* and discharge patients as quickly and safely as possible to the next care setting.

‘There is a huge squeeze of getting people out…we're at max capacity; we need to free up beds…there's a push to get people out. We all feel the squeeze by the medical center “You need to get people out of the hospital.” We're getting pages, getting emails being like, “Get everyone out”’

Hospitalist

*Clinical training and practice norms* were conditions that created normalised care practices. One example of this was that most hospital clinicians had limited medical education or experience in post‐acute care settings. This impacted their ability to advise and set expectations with patients about SNFs.

‘It is humble recognition that for myself, I've spent two days in my career in a SNF—because it's not been provided during training. It sounds a little bit crazy but one of the core things we do as a hospitalist, that I think means so much to us, is how we ensure safe transitions. Yet many of us have never had training or experience working in a SNF.…we send patients out of the hospital but many of us have not had experience in outpatient care’.

Hospitalist

### 3.6 Clinician Processes and Actions

There were five key processes and actions of clinicians. The conditions previously described either independently or, in some instances, in combination, shaped these processes and actions.

#### 3.6.1 Clinician Perceptions of Their Role

Team‐based care and clinical practice norms reinforced clinicians' perceptions of their professional role, particularly around who was responsible or not responsible for preparing patients and discharge and admission to an SNF. Many hospital physicians and nurses felt that setting expectations and counselling patients about the SNF and their recovery was not their professional responsibility.

‘It's not that I've never thought about it. It's just that it's not something that I've ever thought about as being as part of my sort of role in a sense….’

Hospitalist

Another hospitalist was more equivocal:

‘I don't think there's a single person, but I think the majority lays on the case manager who has a little bit more nuanced view as to what places look like, the quality of places, and whether families have liked places or not before in the past. I think the case manager takes the brunt of it, and then the physician takes the medical side of it.’

Hospitalist

#### 3.6.2 Checklist Driven Care and Standardised Workflows

The use of checklists and standardised workflows was normalised and used for almost every part of discharge planning and admission to an SNF. Each hospital day began with a pre‐rounding checklist of clinical topics. Once this was completed, the attending, resident physicians plus medical students would ‘Run the list’ together.

‘Running the list means starting at the top of the list and discussing each patient sequentially, one at a time. We make sure to go over the plan for each patient, discuss what has changed, learn what has been “checked off”, and decide what needs to be added to the list.’

Hospitalist

Another checklist driving care was the patient problem list, which is a list of a patient's medical and social problems. However, problem lists were derived from the clinicians' perspective, meaning they did not include patient‐reported topics or priorities. The use of checklists was common for all clinicians. As described by a nurse, the hospital nurse‐to‐SNF nurse handoff involved a standardised approach that noticeably focused on medical and clinical topics.

‘I typically start with generic information about the patient—allergies, code status, when they were admitted, why they were admitted; a brief course of the patient, like this was the problem …this is how we've been managing them. Then towards the end, I try to go more specific to rehab stuff…updates from physical therapy, what's their mobilization status, how are they eating, what's their diet, when was their last bowel movement, how they use the restroom, are they a fall risk. Then I make sure that I answer any of their questions because sometimes they have a sheet that they're filling out’

Hospital nurse

#### 3.6.3 Creating Medical Stability

The creation of medical stability was also influenced by all three clinician conditions. A hospitalist's primary role through their training was to address a patient's acute illness so they can be discharged. In contrast, health systems wanted this process to be as efficient as possible in freeing up beds for new patients. SNF physicians recognised that the hospital teams focused on medical stability but suggested that this only addressed some factors impacting a patient's recovery and the information that they needed.

‘I am fascinated in how the medical side is always just the top of the iceberg. By the time they come to a post‐acute setting, that tip has already been managed ad nauseam. There are still issues that inevitably arise. My job is to stitch together care in this very vague and nebulous area and have an approach, or an understanding, of what's possible in post‐acute care’.

SNF physician

The prioritisation of medical stability during the preparation for discharge illustrated why patients were so confident in recounting their medical issues but not confident about what SNF care and recovery would look like, given this was not often discussed.

#### 3.6.4 Communication Gaps

Team‐based care resulted in gaps in communication about which clinical tasks were completed and why they were completed. Communication gaps were exacerbated due to throughput pressures.

‘Sometimes it feels like the medical team have no idea how their patients are doing functionally. They just want to know what rehab recommends. They don't really know why we're recommending. It feels like we work in silos ‐ the medical team is managing them medically, we're managing them physically and functionally and nursing is doing everything. Being more aware of a patient's function—and what that means to the patient—is important, because the medical and the functional are so intertwined…’

Physical therapist

To address gaps in team communication, multidisciplinary rounds (MDRs) were held daily during the patient's hospitalisation. MDR was a time for care team members to discuss care and discharge planning. Notably, rarely were all members of the care team present at MDR due to the feasibility of scheduling and competing clinical demands. Physicians and case managers who typically attended MDR acknowledged that patients' individual needs and concerns were usually not discussed in detail.

‘MDR is 10 minutes, and teams can have from 6 to 14 patients. The nitty‐gritty details of how the transition is going to go for a patient, what matters most, what their preferences are—you might hear quick one‐liners, 30 seconds, one minute, about it. But MDR is about running through, patient‐by‐patient, high‐level plans.’

Hospitalist

Nurses also identified morning rounds as a missed opportunity for team communication. This resulted in nurses being unable to complete key parts of their role, which was to provide bedside education, counselling or reinforcement of elements of the transition planning.

‘It's difficult because we don't have it set up to where we do rounds together. I try to keep an eye out for when the team comes so that I can round with them, so that I can be part of that process. But most of the time, the physicians come by in rounds, and we don't even know that they've been there. So, we miss out on those conversations with them and the families’.

Hospital nurse

#### 3.6.5 Recovery Arc Expectation Setting

There is a lack of expectation about recovery by hospital clinicians to patients and caregivers. They acknowledged they did not feel qualified to do so. Hospital clinicians identified gaps in their training and clinical knowledge as reasons why they failed to inform patients about care at SNFs or long‐term recovery. Specifically, their training and focus on inpatient care meant they did not follow patients longitudinally to be able to see the range of potential patient outcomes following hospitalisation. This lack of a feedback loop impacted their confidence in providing prognosis predictions about patients' recovery arc and outcomes.

‘We learn these patterns over time after seeing hundreds and thousands of patients. But if you're a hospitalist your thousands of patients with a hip fracture ends at the time that you discharge them. You don't have that repetition of seeing patterns of people one week, six weeks, three months, one year. Training is lacking in that way. They're missing a part of the pattern. They're understanding how to manage this short time in someone's life, which is sometimes just days in the hospital, but they don't understand the impact of their decisions on long‐term outcomes because they're just not seeing those patterns’.

Hospital geriatrician

### 3.7 Patient and Caregiver Consequences

Four consequences were experienced by patients and caregivers because of the context, conditions, processes and actions of patients, caregivers and clinicians.

#### 3.7.1 Experiencing Uncertainty and Feeling Unprepared for the SNF Admission and Recovery

Feeling uncertain and unprepared was common. When asked to reflect on their discussions with the hospital care team about their SNF admission, some patients and caregivers stated that they received little, if any, information. For others, SNF information was of a general or non‐specific nature.

‘You know, it's funny because we talked about it during hospitalization—when we talked about it, the language…one of the things I remember specifically is that they use a lot of words like, “oh, they're good” or “I've heard they're great.” It's not a lot of context as to how they are or what happens once you are admitted’

Patient

Patients and caregivers lacked knowledge about what to expect in terms of the SNF care they would receive. The ‘step down in care’ that existed between a hospital and an SNF was surprising and concerning. An SNF nurse reflected on this mismatch of expectations:

‘This goes back to the expectation setting. For many of the patients it's their first time at SNF. Patients come thinking that it's going to be like a hospital and that's not the case. Patients may think that they'll be seen more often. Whereas inpatients are often seen daily by the team that's taking care of them, here, our medical providers follow‐up once weekly. If there is an acute issue they are seen more frequently’

SNF nurse

#### 3.7.2 Lacking Control and Agency

Patients reflected that the decision about SNF placement was largely out of their hands. This impacted their sense of control: ‘*You're in somebody's hands. Things are just totally out of your control.’* Even though patients recalled being given a list of SNFs to review, the prospect of choice was false. They really did not have a choice in which SNF they would go to, as this was dictated by insurance and bed availability. For others, when choices were available based on better insurance coverage, they felt rushed in making an important life decision.

‘There is not a lot of time to make that decision. We got a call in the morning, and I got the list maybe an hour later. You must decide quickly, and that is something I think there should at least be a business day between the information getting to the family and the decision’

Caregiver

#### 3.7.3 Wanting Time With the Healthcare Team

Patients and caregivers wanted more time with clinicians. This was partly to help them prepare and understand post‐acute care and their recovery potential, but it was also about having individual connections with their care team. The sentiments of one patient captured how many felt; ‘No one has the time to sit and talk with you in the way that you would want them to’. Another patient noted that the hospital team ‘were talking to each other, but not to me.’ Poor communication resulted in feelings of disconnection between patients and their care team.

#### 3.7.4 Limited Recall of Information

Patients and caregivers commonly reported that they could not recall information given to them. Many challenged the notion that information was even provided, and most noted that information was not forthcoming about what to expect at an SNF or their recovery prognosis and outcomes.

## 4 Discussion

This grounded theory study provides new evidence regarding the drivers and factors that contributed to patients and caregivers suboptimal care experiences during the hospital‐to‐SNF care transition. Our study extends our understanding of these consistently reported experiences by uncovering the dynamic relationships between distinct contextual factors and conditions that shape key processes and actions of patients, caregivers and clinicians that ultimately influence these experiences. Most research investigating the hospital‐to‐SNF care transition has explored the perspective of one key informant group, typically from one care setting. In contrast, this current study has included patients, caregivers and clinicians from both hospitals and SNFs, and therefore, it accurately reflects the combined influences of all informants on this care transition. Using dimensional analysis, we have created an explanatory matrix that has theoretical implications given the conceptualisation of the root causes of problems within this common and significant care transition (Figure 1). This theory generation identifies targets for clinical practice improvement, tailored intervention development and medical education innovations. This has practical implications for improvement efforts, which we will now discuss.

Team‐based care was a condition that had significant implications on clinicians' practice and patient processes. Team‐based care can meet value‐based care goals, reduce clinician burnout and improve efficiency, safety, quality and patient satisfaction [37]. However, despite these benefits, the hospital‐to‐SNF transition created confusion and increased the likelihood of communication failings. As with other studies of hospitalised patients [38, 39], we found that patients were unaware of who was responsible for leading their care and providing them with key information. While efforts have focused on improving understanding of their care team members' names and professional roles [40], this does not fully address the problem. Patients and caregivers in our study misunderstood the fundamental premise of a team care model whereby care, information and support could be delivered by any care team member, not just physicians. Strategies to change how patients perceive and interpret team‐based care are required to clearly articulate, and that is demonstrated in practice, a flatter and less hierarchical organisational structure that supports the intended goals of team‐based care.

Creating processes to optimally deliver information to patients by care teams will only solve part of the knowledge gap experienced by patients. The content of information must also be tailored to a patient's individual needs and circumstances. However, addressing information needs would present challenges based on our results and others which have found that most hospital clinicians, by their own admission, lacked sufficient knowledge about post‐acute SNF care to adequately advise patients and set realistic expectations about potential recovery trajectories [41, 42]. Our study adds to the urgent calls to improve clinical education, training and professional development for hospital‐based clinicians on post‐acute care [43]. A novel finding from our study was that clinicians, regardless of the clinical setting, found it challenging to discuss patients' potential post‐hospital and post‐SNF rehabilitation prognosis, function and outcomes. Prognostic models are emerging that predict functional and other outcomes for patients moving from the hospital to post‐acute care and beyond [44, 45]; however, the implementation and clinical utility of these in real‐world practice settings remains uncertain. How prognostic models can inform real‐time counselling of patients about their long‐term outcomes is a potential avenue for future research.

Patients and caregivers were desperate for more time with their care teams. There is increasing evidence that patients want their care team to know more about them, given they believe it would enhance the patient–clinician relationship, support trust building, improve decision‐making and satisfaction and result in better care and communication [46, 47]. However, studies have repeatedly described that hospital and SNF clinicians spend less time on direct patient care compared to indirect care activities [48, 49, 50]. During transitional care, clinicians must complete indirect clinical and administrative tasks, while the demands on their time are stretched due to high censuses and throughput demands. Care redesign supported by policy and payment incentives that encourage time spent with patients is a challenging suggestion to recommend, but one that has the potential to address many of the drivers of patients' poor experiences during this care transition.

The disconnect between patients, caregivers and care team members was partly a result of the pervasive use of checklists and standardised workflows. Checklists can be a solution to patient safety and quality issues; however, they can also fail to account for and address complex, challenging healthcare situations [51]. The hospital‐to‐SNF transition is one example where a checklist appears to exacerbate problems rather than solve them. The use of checklists created a clinical task‐oriented environment that did not allow for tasks or a checkbox to elicit or address patients' individual transition and recovery priorities. Creating opportunities for patients and caregivers to be partners in this transition may offer solutions. One potential approach could be the development of SNF care transition interventions that engage, empower and educate patients. Interventions to engage patients and support goal setting, values elicitation and skill building for communication may also offer a solution to several of the topics identified in this study [52, 53].

Our study has several limitations. First, the geographic restriction to one AMC and SNF in one city may limit transferability to other settings. Second, it only included English speakers and people without ADRD—these patient populations should be considered in future research. Third, while the research team's clinical expertise may have enriched the data and analysis, our positionality as clinicians and researchers may have influenced findings in unrecognised ways.

## 5 Conclusions

Our study provides new evidence regarding the drivers and factors that contribute to patients' and caregivers' suboptimal care experiences during the hospital‐to‐SNF care transition. Using dimensional analysis, we have created an explanatory matrix that has theoretical implications given the conceptualisation of the root causes of problems within this common and significant care transition. This theory generation identifies targets for clinical practice improvement, tailored intervention development and medical education innovations.

## Author Contributions

**James D. Harrison:** conceptualization, investigation, funding acquisition, writing – original draft, visualization, validation, writing – review and editing, formal analysis, project administration, data curation, methodology. **Margaret C. Fang:** conceptualization, investigation, funding acquisition, writing – review and editing, visualization, validation, resources, supervision, data curation. **Rebecca L. Sudore:** conceptualization, funding acquisition, investigation, validation, visualization, writing – review and editing, supervision, data curation. **Andrew D. Auerbach:** conceptualization, funding acquisition, validation, visualization, writing – review and editing, supervision, data curation. **Tasce Bongiovanni:** data curation, investigation, visualization, validation, writing – review and editing, formal analysis. **Audrey Lyndon:** supervision, methodology, conceptualization, investigation, funding acquisition, validation, visualization, writing – review and editing, formal analysis, resources, data curation.

## Ethics Statement

This study was approved by the University of California San Francisco (UCSF) Institutional Review Board (approval #21‐33596).

## Consent

As approved by the UCSF IRB, all participants provided written informed consent for interviews and verbal informed consent for observations.

## Conflicts of Interest

The authors declare no conflicts of interest.

## Supporting information

Appendix 1.

Appendix 2.

Appendix 3.

## Data Availability

De‐identified study data is available upon request from the corresponding author.

## References

[1] Medicare Payment Advisory Commission. Medicare Payment Policy: Report to Congress. Medicare Payment Advisory Commission, 2024, https://www.medpac.gov/document/march-2024-report-to-the-congress-medicare-payment-policy/.

[2] Medicare.gov. Skilled nursing facility care, accessed January 17, 2025, https://www.medicare.gov/coverage/skilled-nursing-facility-care.

[3] Definitive Healthcare. What is a skilled nursing facility (SNF)?, accessed January 17, 2025, https://www.definitivehc.com/resources/glossary/skilled-nursing-facility.

[4] R. E. Burke , E. Juarez‐Colunga , C. Levy , A. V. Prochazka , E. A. Coleman , and A. A. Ginde , “Patient and Hospitalization Characteristics Associated With Increased Postacute Care Facility Discharges From US Hospitals,” Medical Care 53, no. 6 (2015): 492–500, 10.1097/MLR.0000000000000359. 25906015 PMC4431940

[5] S. L. Feder , M. C. Britton , and S. I. Chaudhry , “‘They Need to Have an Understanding of Why They're Coming Here and What the Outcomes Might Be.’ Clinician Perspectives on Goals of Care for Patients Discharged From Hospitals to Skilled Nursing Facilities,” Journal of Pain and Symptom Management 55, no. 3 (2018): 930–937, 10.1016/j.jpainsymman.2017.10.013. 29097273

[6] B. J. King , A. L. Gilmore‐Bykovskyi , R. A. Roiland , B. E. Polnaszek , B. J. Bowers , and A. J. Kind , “The Consequences of Poor Communication During Transitions From Hospital to Skilled Nursing Facility: A Qualitative Study,” Journal of the American Geriatrics Society 61, no. 7 (2013): 1095–1102, 10.1111/jgs.12328. 23731003 PMC3714367

[7] M. C. Britton , G. M. Ouellet , K. E. Minges , M. Gawel , B. Hodshon , and S. I. Chaudhry , “Care Transitions Between Hospitals and Skilled Nursing Facilities: Perspectives of Sending and Receiving Providers,” Joint Commission Journal on Quality and Patient Safety 43, no. 11 (2017): 565–572, 10.1016/j.jcjq.2017.06.004. 29056176 PMC5693352

[8] E. A. Gadbois , D. A. Tyler , R. Shield , et al., “Lost in Transition: A Qualitative Study of Patients Discharged From Hospital to Skilled Nursing Facility,” Journal of General Internal Medicine 34, no. 1 (2019): 102–109, 10.1007/s11606-018-4695-0. 30338471 PMC6318170

[9] P. A. Valverde , R. Ayele , C. Leonard , E. Cumbler , R. Allyn , and R. E. Burke , “Gaps in Hospital and Skilled Nursing Facility Responsibilities During Transitions of Care: A Comparison of Hospital and SNF Clinicians' Perspectives,” Journal of General Internal Medicine 36, no. 8 (2021): 2251–2258, 10.1007/s11606-020-06511-9. 33532965 PMC8342702

[10] R. E. Burke , J. Jones , E. Lawrence , et al., “Evaluating the Quality of Patient Decision‐Making Regarding Post‐Acute Care,” Journal of General Internal Medicine 33, no. 5 (2018): 678–684, 10.1007/s11606-017-4298-1. 29427179 PMC5910345

[11] B. W. Clark , K. Baron , K. Tynan‐McKiernan , M. C. Britton , K. E. Minges , and S. I. Chaudhry , “Perspectives of Clinicians at Skilled Nursing Facilities on 30‐Day Hospital Readmissions: A Qualitative Study,” Journal of Hospital Medicine 12, no. 8 (2017): 632–638, 10.12788/jhm.2785. 28786429

[12] K. L. Ryskina , K. A. Foley , J. H. Karlawish , et al., “Expectations and Experiences With Physician Care Among Patients Receiving Post‐Acute Care in US Skilled Nursing Facilities,” BMC Geriatrics 20, no. 1 (2020): 463, 10.1186/s12877-020-01869-1. 33172392 PMC7653446

[13] C. Levine and K. Ramos‐Callan , The Illusion of Choice: Why Decisions About Post‐Acute Care Are Difficult for Patients and Family Caregivers (United Hospital Fund, 2019).

[14] P. Kothari and J. Guzik , Health Care Provider Perspectives on Discharge Planning: From Hospital to Skilled Nursing Facility (United Hospital Fund, 2019).

[15] E. A. Gadbois , D. A. Tyler , and V. Mor , “Selecting a Skilled Nursing Facility for Postacute Care: Individual and Family Perspectives,” Journal of the American Geriatrics Society 65, no. 11 (2017): 2459–2465, 10.1111/jgs.14988. 28682444 PMC5681373

[16] L. Rogut and A. J. Aude *. Pathways to Progress on Difficult Decisions in Post‐Acute Care*. 2019, https://uhfnyc.org/media/filer_public/f6/91/f691d584-53d0-4056-ba0a-99d599d2a446/diffdecisions4-20190313.pdf.

[17] U.S. Department of Health and Human, Services, Centers for Medicare & Medicaid Innovation, Center for Medicare and Medicaid Group L and D. Defining and Using Aims and Drivers for Improvement. 2013, https://www.cms.gov/priorities/innovation/files/x/hciatwoaimsdrvrs.pdf.

[18] L. Herndon , C. Bones , P. Bradke , and P. Rutherford. *How‐to Guide: Improving Transitions From the Hospital to Skilled Nursing Facilities to Reduce Avoidable Rehospitalizations*. Institute for Healthcare Improvement. 2013.

[19] National Quality Forum. Effective Communication and Care Coordination. 2020, accessed August 9, 2024, https://www.qualityforum.org/Topics/Effective_Communication_and_Care_Coordination.aspx.

[20] Centers for Medicare and Medicaid Services. National Care Transitions Awareness Day Promotes Safe, Effective, Person‐Centered Care. 2019, accessed December 5, 2021, https://www.cms.gov/blog/national-care-transitions-awareness-day-promotes-safe-effective-person-centered-care.

[21] J. D. Harrison , M. Archuleta , E. Avitia , et al., “Developing a Patient‐ and Family‐Centered Research Agenda for Hospital Medicine: The Improving Hospital Outcomes Through Patient Engagement (i‐HOPE) Study,” Journal of Hospital Medicine 15, no. 6 (2020): 331–337, 10.12788/jhm.3386. 32490806 PMC7289507

[22] K. Charmaz , Constructing Grounded Theory, 1st ed. (Sage, 2006).

[23] H. Blumer , Symbolic Interactionism: Perspective and Method (University of California Press, 1969).

[24] Hospital Medicine Reengineering Network (HOMERuN). HOMERuN Patient & Family Advisory Council (PFAC). 2024, accessed June 25, 2024, https://hospitalinnovate.org/about-homerun/patient-family-advisory-council/.

[25] M. Hammersley and P. Atkinson , Ethnography: Principles in Practice (Routledge, 2007), 10.4324/9781315146027.

[26] M. Dixon‐Woods and C. Bosk , “Learning through Observation: The Role of Ethnography in Improving Critical Care,” Current Opinion in Critical Care 16, no. 6 (2010): 639–642, https://journals.lww.com/co-criticalcare/Fulltext/2010/12000/Learning_through_observation__the_role_of.21.aspx. 20808219 10.1097/MCC.0b013e32833ef5ef

[27] E. L. Wanko Keutchafo , J. Kerr , and M. A. Jarvis , “Evidence of Nonverbal Communication Between Nurses and Older Adults: A Scoping Review,” BMC Nursing 19, no. 1 (2020): 53, 10.1186/s12912-020-00443-9. 32550824 PMC7298765

[28] M. Birks , Y. Chapman , and K. Francis , “Memoing in Qualitative Research: Probing Data and Processes,” Journal of Research in Nursing 13, no. 1 (2008): 68–75, 10.1177/1744987107081254. PMC12602296 41229605

[29] N. H. Wolfinger , “On Writing Fieldnotes: Collection Strategies and Background Expectancies,” Qualitative Research 2, no. 1 (2002): 85–93, 10.1177/1468794102002001640.

[30] L. Schatzman , “Dimensional Analysis: Notes on an Alternative Approach to Grounded Theory in Qualitative Research.” In Social Organization and Social Process: Essays in Honor of Anseim Strauss, ed. D. Maines (Aldine De Gruyer, 1991), 303–314.

[31] S. Kools , M. McCarthy , R. Durham , and L. Robrecht , “Dimensional Analysis: Broadening the Conception of Grounded Theory,” Qualitative Health Research 6, no. 3 (1996): 312–330, 10.1177/104973239600600302.

[32] K. Wisner , C. A. Chesla , J. Spetz , and A. Lyndon , “Managing the Tension Between Caring and Charting: Labor and Delivery Nurses' Experiences of the Electronic Health Record,” Research in Nursing & Health 44, no. 5 (2021): 822–832, 10.1002/nur.22177. 34402080

[33] W. G. Anderson , S. Kools , and A. Lyndon , “Dancing Around Death: Hospitalist‐Patient Communication About Serious Illness,” Qualitative Health Research 23, no. 1 (2013): 3–13, 10.1177/1049732312461728. 23034778 PMC3502664

[34] G. A. Bowen , “Grounded Theory and Sensitizing Concepts,” International Journal of Qualitative Methods 5, no. 3 (2006): 12–23, 10.1177/160940690600500304.

[35] S. J. Tracy , “Qualitative Quality: Eight ‘Big‐Tent’ Criteria for Excellent Qualitative Research,” Qualitative Inquiry 16, no. 10 (2010): 837–851, 10.1177/1077800410383121.

[36] R. Whittemore , S. K. Chase , and C. L. Mandle , “Validity in Qualitative Research,” Qualitative Health Research 11, no. 4 (2001): 522–537, 10.1177/104973201129119299. 11521609

[37] K. K. Will , M. L. Johnson , and G. Lamb , “Team‐Based Care and Patient Satisfaction in the Hospital Setting: A Systematic Review,” Journal of Patient‐Centered Research and Reviews 6, no. 2 (2019): 158–171, 10.17294/2330-0698.1695. 31414027 PMC6676761

[38] M. K. Atkinson , M. Wazir , E. Barkoudah , et al., “Inpatient Understanding of Their Care Team and Receipt of Mixed Messages: A Two‐Site Cross‐Sectional Study,” Journal of General Internal Medicine 38, no. 12 (2023): 2703–2709, 10.1007/s11606-023-08178-4. 36973573 PMC10042424

[39] N. Curatola , N. Juergens , M. K. Atkinson , et al., “Inpatients' Understanding of the Hospitalist Role and Common Medical Terminology,” Journal of Hospital Medicine 20, no. 1 (2025): 51–55, 10.1002/jhm.13492. 39199015 PMC11866783

[40] V. M. Arora , C. Schaninger , M. D'arcy , et al., “Improving Inpatients' Identification of Their Doctors: Use of FACE Cards,” Joint Commission Journal on Quality and Patient Safety 35, no. 12 (2009): 613–619. 20043501 10.1016/s1553-7250(09)35086-2 PMC3188440

[41] P. A. Valverde , R. Ayele , C. Leonard , E. Cumbler , R. Allyn , and R. E. Burke , “Gaps in Hospital and Skilled Nursing Facility Responsibilities During Transitions of Care: A Comparison of Hospital and SNF Clinicians' Perspectives,” Journal of General Internal Medicine 36, no. 8 (2021): 2251–2258, 10.1007/s11606-020-06511-9. 33532965 PMC8342702

[42] R. E. Burke , E. Lawrence , A. Ladebue , et al., “How Hospital Clinicians Select Patients for Skilled Nursing Facilities,” Journal of the American Geriatrics Society 65, no. 11 (2017): 2466–2472, 10.1111/jgs.14954. 28682456 PMC5681432

[43] C. D. Jones and R. E. Burke , “Annals for Hospitalists Inpatient Notes—Getting Past the ‘Black Box’—Opportunities for Hospitalists to Improve Postacute Care Transitions,” Annals of Internal Medicine 168, no. 10 (2018): HO2–HO3, 10.7326/M18-0940. PMC6592605 29800449

[44] A. A. Esteban‐Burgos , J. El Mansouri‐Yachou , R. Muñoz‐Ramirez , C. Hueso‐Montoro , M. P. Garcia‐Caro , and R. Montoya‐Juarez , “Prognostic Models Associated With 6‐Month Survival of Patients Admitted to Nursing Homes,” Gerontology 65, no. 1 (2019): 40–44, 10.1159/000490243. 29961071

[45] R. E. Burke , E. Hess , A. E. Barón , C. Levy , and J. D. Donzé , “Predicting Potential Adverse Events During a Skilled Nursing Facility Stay: A Skilled Nursing Facility Prognosis Score,” Journal of the American Geriatrics Society 66, no. 5 (2018): 930–936, 10.1111/jgs.15324. 29500814 PMC5992035

[46] D. L. Zimmerman , D. J. Min , A. Davis‐Collins , and P. DeBlieux , “Treating Patients as People: What Do Hospital Patients Want Clinicians to Know About Them as a Person?,” Journal of Patient Experience 7, no. 2 (2020): 270–274, 10.1177/2374373519826244. 32851151 PMC7427369

[47] P. Evans , B. Rogers , G. Symczak , et al., “Earn Our Trust: The Perspectives of Patients and Caregivers,” Journal of Hospital Medicine 17 (2022): 313–315, 10.1002/jhm.12796. 35535930 PMC9096919

[48] K. H. Chaiyachati , J. A. Shea , D. A. Asch , et al., “Assessment of Inpatient Time Allocation Among First‐Year Internal Medicine Residents Using Time‐Motion Observations,” JAMA Internal Medicine 179, no. 6 (2019): 760–767, 10.1001/jamainternmed.2019.0095. 30985861 PMC8462976

[49] O. Michel , A. J. Garcia Manjon , J. Pasquier , and C. Ortoleva Bucher , “How Do Nurses Spend Their Time? A Time and Motion Analysis of Nursing Activities in an Internal Medicine Unit,” Journal of Advanced Nursing 77, no. 11 (2021): 4459–4470, 10.1111/jan.14935. 34133039 PMC8518809

[50] Y. J. Kang , C. A. Mueller , J. E. Gaugler , M. A. Mathiason Moore , and K. A. Monsen , “Toward Ensuring Care Quality and Safety Across Settings: Examining Time Pressure in a Nursing Home With Observational Time Motion Study Metrics Based on the Omaha System,” Journal of the American Medical Informatics Association 30, no. 11 (2023): 1837–1845, 10.1093/jamia/ocad113. 37352394 PMC10586029

[51] K. Catchpole and S. Russ , “The Problem With Checklists,” BMJ Quality & Safety 24, no. 9 (2015): 545–549, 10.1136/bmjqs-2015-004431. 26089207

[52] S. E. Hickman , H. D. Lum , A. M. Walling , A. Savoy , and R. L. Sudore , “The Care Planning Umbrella: The Evolution of Advance Care Planning,” Journal of the American Geriatrics Society 71, no. 7 (2023): 2350–2356, 10.1111/jgs.18287. 36840690 PMC10958534

[53] R. D. McMahan , S. E. Hickman , and R. L. Sudore , “What Clinicians and Researchers Should Know About the Evolving Field of Advance Care Planning: A Narrative Review,” Journal of General Internal Medicine 39, no. 4 (2024): 652–660, 10.1007/s11606-023-08579-5. 38169025 PMC10973287

