# Supplementary material for: ‘They Were Talking to Each Other but Not to Me’: Examining the Drivers of Patients' Poor Experiences During the Transition From the Hospital to Skilled Nursing Facility
Source: Health Expect. 2025 Apr 28;28(3):e70248. doi: 10.1111/hex.70248 (PMC12037702; doi:10.1111/hex.70248)
Supplement: Supplementary file 1 — Appendix 1. [file HEX-28-e70248-s003.docx]

Appendix 1: Details about study sites and the care delivered.

University of California, San Francisco (UCSF) - **Academic Medicine Center**

Located in the U.S., the University of California, San Francisco (UCSF) is a leading academic medical center, meaning it is a hospital associated with UCSF Medical school. As both a research and teaching hospital, faculty of UCSF are not only involved in caring for patients but also in teaching the next generation of healthcare professionals and conducting innovative research improving the quality and delivery of healthcare.

Skilled nursing facility (SNF)

A skilled nursing facility (SNF) is an inpatient healthcare facility with round-the-clock nursing services. Some SNFs provide short term care in the form of acute rehabilitation, e.g., helping an adult who has recently been discharged from the hospital following knee surgery develop the necessary strength and walking abilities before moving home. Other SNFs provide long-term care and permanently house patients who have ongoing nursing and support needs.

San Francisco Campus for Jewish Living (SFCJL)

The San Francisco Campus for Jewish Living (SFCJL) is a senior health services organization offering many levels of care. SFCJL comprises an acute rehabilitation SNF as well as several long-term care facilities providing various levels of health and nursing support, ranging from a nursing home to assisted living and memory care unit for folks with cognitive impairment and dementia.
