# Supplementary material for: ‘They Were Talking to Each Other but Not to Me’: Examining the Drivers of Patients' Poor Experiences During the Transition From the Hospital to Skilled Nursing Facility
Source: Health Expect. 2025 Apr 28;28(3):e70248. doi: 10.1111/hex.70248 (PMC12037702; doi:10.1111/hex.70248)
Supplement: Supplementary file 2 — Appendix 2. [file HEX-28-e70248-s002.docx]

# **Patient & Caregiver Interview Guide**

Can you tell me about yourself and why you were in the hospital recently?

Can you talk me through why you (or the person you are caregiving for) has come to the skilled nursing facility?

How have you been since you (or the person you are a caregiver for) has left the hospital?

Can you tell me about the move/ from the hospital to this skilled nursing facility?

- - What went well?
  - What parts were difficult?
  - Can you tell me about any conversations you had care team in the hospital?
  - Can you tell me about any conversations you had care team in the skilled nursing facility?

How were you prepared by the hospital care team before coming to the skilled nursing facility?

Now that you are here at the skilled nursing facility, can you tell me about anything the hospital team could have done better to help prepare for you being here?

Can you tell me about any remaining concerns that you have following your hospitalization and recovery?

Do you have any suggestions about how the hospital to care transition can be improved?

Think back to the time before you were in hospital and your typical day. Talk me through what your day looked like, what type of activities were meaningful to you? What did you enjoy and/or do for fun?

Can you tell me about what matters most to you now (or the person you are caregiver for) in terms of your recovery?

- - Can you tell me about the time when your healthcare team talked to you about what matters most?

**Care Team Interview Guide**

- Can you tell me about yourself and your professional role?
- Thinking back to the last person you transitioned to skilled nursing
  - Tell me about that person.
  - What was the transition process like? Describe it to me.
- When you are preparing to transfer a patient to skilled nursing, what are your priorities?
  - Can you talk me through your decision-making process when you are deciding whether a patient needs to go to a SNF? Can you provide a specific case as an example?
- How do you set plans with the patient/family?
  - How are patients and their families prepared for a hospital to SNF discharge/transition?
  - Can you tell me about the information you elicit from patients and families as you consider placing them in skilled nursing facility?
- Can you talk me through what you expect will happen to patients once a patient has left the hospital and are admitted to a SNF for post-acute care?
- Can you tell me about a time when the hospital to SNF transition was difficult?
  - What happened?
  - In your perspective, how can the hospital to SNF transition be improved?
- Is there anything else you would like to add related to the hospital to SNF care transition?
- How do you identify what matters most to the patient and their families? Can you give me an example?
  - Who is responsible for incorporating what matters most into patients care transition plans?
  - What do the terms patient preferences and patient values mean to you?
