# Supplementary material for: ‘They Were Talking to Each Other but Not to Me’: Examining the Drivers of Patients' Poor Experiences During the Transition From the Hospital to Skilled Nursing Facility
Source: Health Expect. 2025 Apr 28;28(3):e70248. doi: 10.1111/hex.70248 (PMC12037702; doi:10.1111/hex.70248)
Supplement: Supplementary file 3 — Appendix 3. [file HEX-28-e70248-s001.docx]

**Appendix 3: Selected Additional representative quotes and observation field notes.**

**Patients, caregivers and clinicians**

**Context**

***Healthcare settings***

The Medicine Service at this academic medical center is the largest admitting service and includes eight care teams (comprising hospitalist physicians, resident physicians, and students) and 10 direct care hospitalist-only services. The average daily census for the Medicine Service is N patients with over 8000 patients admitted annually. Patients who are admitted to the medicine service and hospital from the emergency room are acutely unwell. There is no single diagnosis that accounts for admissions but rather a range of diagnosis, chronic conditions, and behavioral conditions. In addition to physicians, patients receive care from the interdisciplinary clinical team including nurses, case managers, social workers, physical and occupational therapists

The SNF is a rehabilitation facility with approximately 90 beds for temporary short-term stays of ≤100 days. To be eligible for admission, patients must have had a previous 3-day stay at an acute hospital. For this study, patients were included if they are recovering from a primary acute medical illness. SNF care includes comprehensive medical rehabilitation including medical management by on-site physicians (internists, hospitalists, or geriatricians), licensed nursing care, physical, occupational and speech therapies. A clinical affiliation between the academic medical center and SNF has been in operation since 2008. Around 500 patients from the academic center are admitted to this SNF annually.

***A life changing accident or chronic illness***

For patient participants, the context for their hospitalization and admission to a SNF was a life changing event. For example, it was an accident; a fall in a bathroom, being knocked over due to high winds, or a knee giving up resulting in a fall after waiting in-line for hours at the social security administration. For others it was an acute exacerbation of a chronic illness such as congestive heart failure, a persistent infection, , breathing issues related to chronic obstructive pulmonary disease or an urgent need to control their diabetes. Hospitalization then created, or exacerbated pre-existing, functional limitations that led to deconditioning and decline that resulted in the need for post-acute care rehabilitation.

***Physical and emotional vulnerability***

*“It’s another day for us and it’s what we do every day, but there’s no taking away that for many of the patients and their families, being admitted to the hospital…it’s the worst days of their life. I think that there’s a weight to that.” (hospitalist)*

Often, the only thing personalizing a room was the patient’s name written on the door. Personal belongings were left in plastic bags or stacked on bedside tables. The main feature of patients’ room in both settings were televisions, often on loudly to distract them from their boredom, worries or symptoms that they were experiencing. Patients spent a lot of time alone except for when care was provided, meals were delivered, or rooms cleaned. Within the SNF, there were several daily social activities that residents could participate, although not all patients participated in these activities. The vulnerability experienced during this care transition added to patients’ feelings of uncertainty and desire for connections with their care team.

**Patients and caregivers**

***Limited Information availability***

To ensure caregivers captured the information the care team discussed, they would use their phones to record conversations. In some instances, caregivers who were often not present during care team interactions would diligently check the electronic patient portal to keep up to date on their loved one’s status and care.

“*I keep diligent notes. I spend a lot of time reading progress notes when she’s hospitalized, like my routine in the morning is I literally log into her MyChart, pull up her visit, and read whatever progress notes have been posted the night before. I do. I read it, I read all of it because sometimes there’s been some things that have been noted in those progress notes that are concerning to me.” (caregiver)*

**Clinicians**

**Conditions**

***Pressure to maintain hospital throughput***

“We all feel the squeeze by the medical center of like, "You need to get people out of the hospital." We're getting pages, getting emails being like, "Get everyone out." It's kind of like if you're given two choices of like, "I'm going to open up this discussion that could potentially lead to maybe some resistance that would delay the discharge or time for the case manager," I can understand how they're like, "I'm going to keep it simple. I'm going to ask them yes or no questions. I'm going to give them these options, and those are the options," rather than keeping it like pretty open for those practical purposes. Do I think that's right? Probably not. But I also can acknowledge the system issues that are real limitations for bandwidth on everyone's end” (hospitalist)

“And the hidden curriculum in hospital medicine is you are rewarded for discharging people quickly. People ask you, "Did you process that discharge?" If it was a difficult discharge, if you managed to pull that off, then you are rewarded for it.” (geriatrician SNF)

**Processes**

***Communication gaps between the care team***

Some communication gaps were due to how information is recorded in the EHR. While there is the potential for the team to access each other’s notes, this was rarely done given how each clinicians notes were organized and separated from each other. This meant elements of care delivered by other team members could be missed as well as the potential for a shared understanding of the priorities of patients during this care transition.

*“Everyone has access to our notes’ now it's a matter if they read it or not. I think sometimes the issue that we have from a therapy perspective is we write so much, and a lot of times it is physical therapy jargon. And so for a lot of people, they are not processing or comprehending what we're actually doing in those PT sessions and why” (physical therapist).*

***Checklist driven care***

Reminders for clinicians in non-patient areas about checklists and standardized workflows were common. For example, posters were found on the walls in resident rooms, on computer monitor screen savers, and in bathrooms. Poster reminders included a variety of checklist or workflow topics - discharge processes, bedside medication delivery, hospital quality goals, admission checklist, resuscitation processes, and advanced care planning documentation.

“So during the day, they gave me this paper. They're coming and saying, "You're going to leave. You need to do this; you need to do that." And this woman came and said, "Here, you need to sign this…So it was just by the book. It has to be done this way. And it's the tone of voice that people use with you, that I have to do it”

***Creating medical stability***

Information provided to patients during hospitalization focused on topics that were prioritized by the clinical team. Specifically, information that related to creating medical stability so that the logistics of discharge planning could commence.

*“My priorities are related to the discharge barriers in terms of the acute medical reason why they've been hospitalized, making sure those things are lined up. Then I'm in communication with case management about the potential need for a skilled nursing facility…so that if we're anticipating a discharge like a day or two out, that the process is already started in terms of referrals, and making sure the family and the patient agree with that…” (hospitalist)*

“So really my focus is on making sure that the medical transition is safe and that all the loose ends are communicated.

**Recovery arc expectation setting**

*“I feel like I'm not as involved in that process as much as I'd like to be, I don't have an expertise in it, so I don't want to misinform people or set unrealistic expectations about places they are going to” (hospital nurse)*

“So the feedback loop -- and that is a different discussion about whether that needs to be part of our training. We send patients out of the hospital to primary care providers or outpatient folks. Many of us have not had experience in outpatient care…we often don't get the close feedback loop, unless we call patients at a SNF -- which I'll share with you, and which and I imagine for most folks we don't do -- or we hear directly from patients when they return back to the hospital”

**Patients consequences and outcomes**

***Experiencing uncertainty and feeling unprepared for the SNF admission and recovery***

*“When I was there in the hospital they told me, “You’re going to the rehab facility in order to get your exercise” …That’s all they told me. Nothing else” (patient)*

Patients and caregivers lacked knowledge about what to expect at a SNF in terms of the care they would receive, specifically regarding the “*step down in care”* that existed between hospital and a SNF. This was surprising and concerning especially for caregivers:

*“So part of me thought I'm going to have a little break from paying my private person but absolutely not. I mean, he needs to have her here. Honestly, part of me was a little bit looking forward to it. My husband's been sick for around eight or 10 years. I'm 17 years younger than him. We're kind of in different places in our life. I thought coming here might be a little bit of a break for me…. I don't feel like I can just relax, and say everything's great…I don't feel that way. I wish I did….It would have been a wonderful bit of respite for me. But I don't feel that way ….”* Caregiver

Information about their post-acute recovery and function was also absent. As one patient noted “*I don't think there was any specific this-is-how-it's-going-to-go-down-the-road kind of thing in terms of my recovery”.*

***Lack of control and agency***

*“Discharge planning came and talked to me. They gave me a list of different places that do it so I could look myself online, read about the facilities. But it came down to who had an opening basically and who would accept me…” (patient)*

For others when choices were available based on better insurance coverage, they felt rushed and lacked agency into making an important life decision.

*“The other thing that I think to consider is that there is not a lot of time to actually make that decision. I mean, my God, they were talking about discharging her! We got a call in the morning, and I got the list maybe an hour later. You must make a decision really quickly, and that is something that I think that there should at least be a business day between the information getting to the family and the decision” (Caregiver)*
